# Protocol for efficient purification of endotoxin-free human recombinant tau protein

**DOI:** 10.1016/j.xpro.2026.104800

**Published:** 2026-08-27

**Authors:** Maria Kreger Karabova, Anna del Ser-Badia, William S. James, Sally A. Cowley

**Affiliations:** 1 James and Lillian Martin Centre for Stem Cell Research, Sir William Dunn School of Pathology, University of Oxford, Oxford OX1 3RE, UK

**Keywords:** Immunology, Protein Biochemistry, Protein expression and purification

## Abstract

Microglia, the brain-resident immune cells, can propagate tau protein in a prion-like manner in neurodegenerative diseases called tauopathies. Here, we present an efficient protocol for the purification of endotoxin-free human recombinant tau protein. We detail steps for tau expression in *E. coli*, followed by on-column Triton X-114-based endotoxin depletion during purification, concentration, residual endotoxin removal, and quality control of the final product. We then describe a workflow for heparin-induced *in vitro* aggregation of purified tau into preformed fibrils. This protocol enables an accurate investigation of the microglial response to tau without the confounding effect of residual endotoxin.

For complete details on the use and execution of this protocol, please refer to Karabova et al.^1^

## Highlights

• Steps for expressing 6xHis-SUMO3-tagged, human recombinant 2N4R tau protein in *E. coli*
• Steps for an efficient endotoxin removal using an on-column Triton X-114 wash
• Instructions for SUMO-tag cleavage, final purification, and product quality control
• Guidance on heparin-induced *in vitro* tau fibril formation and validation

## Abstract (editor highlights)

Publisher’s note: Undertaking any experimental protocol requires adherence to local institutional guidelines for laboratory safety and ethics.

## Graphical abstract

**Figure:**
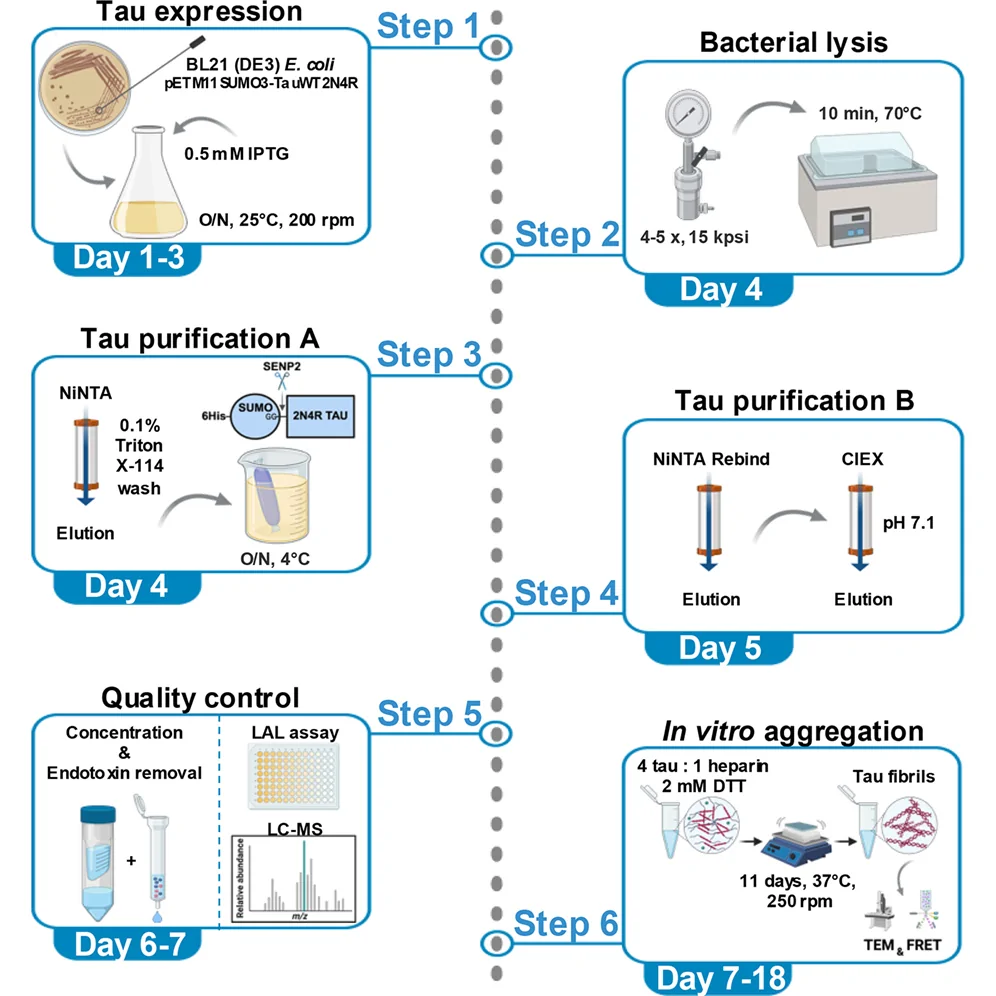

## Before you begin

Recombinant tau protein is widely used to study the mechanisms underlying tau aggregation and its cellular effects in neurodegenerative diseases. However, its use in immunocompetent cell systems, such as microglia, poses a significant methodological challenge due to endotoxin contamination inherent to *E. coli*-based expression systems. Endotoxins, or lipopolysaccharides (LPS), are outer-membrane components of gram-negative bacteria and highly heat and pH stable amphipathic molecules that, once released during bacterial lysis, can strongly bind to expressed proteins - particularly those with a positive net charge, such as tau.^2^^,^^3^^,^^4^ Even trace amounts of endotoxin can elicit a potent inflammatory response in microglia via the CD14-TLR4-MD2 signaling axis, leading to cytokine release independent of tau-specific biological activity.^5^^,^^6^^,^^7^^,^^8^^,^^9^

Because endotoxin removal from recombinant protein preparations is notoriously difficult and often inefficient, many studies investigating microglial responses to recombinant tau have been confounded by unquantified or unaddressed endotoxin contamination, obscuring the distinction between tau-specific and endotoxin-driven effects (reviewed in^1^). A theoretical alternative is the use of ClearColi™ bacterial strains engineered to produce non-immunogenic Lipid IVA in place of the Lipid A recognised by the immunocompetent cells.^10^ However, in our experience, this approach is limited by reduced bacterial growth kinetics and low recombinant tau protein yield, rendering it impractical for high-throughput downstream applications *in vitro*.^11^ Similarly, we found that commercially available endotoxin removal columns alone were insufficient to reduce endotoxin contamination to acceptable levels. This likely reflects the strong electrostatic interactions between negatively charged endotoxin and the net positively charged 2N4R tau, which promote the formation of stable complexes that are resistant to dissociation during purification.^3^^,^^12^ Consistent with this, endotoxin removal has been reported to be less efficient for basic proteins compared with acidic counterparts,^13^ in line with our observation of persistent endotoxin contamination following multiple affinity-based depletion attempts.^11^

Recently, Shultz et al.^14^ have described a protocol for post-purification endotoxin removal from recombinant tau using Triton X-114 phase separation, followed by validation of endotoxin depletion using TLR4 reporter cells and downstream cellular assays. While this approach provides an effective strategy for reducing endotoxin bioactivity in existing recombinant tau preparations, it requires repeated detergent extraction and subsequent detergent removal. To address the broader challenges associated with the production of endotoxin-free recombinant tau, we developed a protocol that integrates endotoxin removal directly into the purification workflow while maintaining high protein purity and yield.

This protocol provides a robust method for the production of endotoxin-free, full-length (2N4R) human recombinant tau protein, suitable for downstream *in vitro* applications involving microglia or other immune-competent cells.

### Innovation

While based on established tau purification methodologies,^15^^,^^16^ this workflow introduces several key innovations that improve both protein purity and endotoxin removal.

Firstly, it combines Ni-NTA affinity chromatography with an on-column depyrogenation wash using 0.1% Triton X-114. Unlike post-purification detergent extraction methods, this approach effectively dissociates endotoxin-tau complexes immediately following protein capture and integrates endotoxin removal directly into the purification workflow, thereby minimizing sample handling while maintaining high protein recovery. Second, the use of a 6xHis-SUMO3 fusion tag reduces proteolytic tau degradation and allows precise tag removal by SENP2 protease, yielding a highly pure tau preparation with a native N-terminus and avoiding the extraneous residues typically introduced by TEV protease cleavage of conventional 6xHis-tagged constructs.^17^^,^^18^^,^^19^ Third, an additional cation-exchange chromatography (CIEX) step is followed by the use of a high-capacity endotoxin removal resin to ensure effective removal of any residual endotoxin in the preparation.

Finally, the protocol includes a simple, scalable and reproducible method for generating *in vitro*-aggregated, seeding-competent tau fibrils from purified monomeric tau, enabling downstream studies of cellular response to disease-relevant tau species. Although optimized for full-length human tau as the predominant tau species found in tauopathy-associated aggregates^20^^,^^21^ the workflow can be readily adapted for purification of other tau isoforms and potentially other positively charged recombinant proteins that are susceptible to endotoxin contamination.

## Key resources table

**Table:** 

REAGENT or RESOURCE	SOURCE	IDENTIFIER
**Bacterial and virus strains**		

BL21(DE3) Competent Cells	Thermo Fisher Scientific	Cat#EC0114

**Chemicals, peptides, and recombinant proteins**		

β-mercaptoethanol (BME)	MP Biomedicals	Cat#190242
Bond-breaker™ TCEP Solution, neutral pH	Thermo Fisher Scientific	Cat#77720
DL-Dithiothreitol (DTT)	Sigma-Aldrich	Cat#43816
DNase I	Invitrogen	Cat#18047-019
Glycerol	Sigma-Aldrich	Cat#G6279
Heparin	Sigma-Aldrich	Cat#H3393
HEPES	Sigma-Aldrich	Cat#H3375-500G
Hydrochloric acid 37%	VWR Chemicals	Cat#7647-01-0
Imidazole	Millipore	Cat#288-32-4
IPTG	Sigma-Aldrich	Cat#I5502-5G
Kanamycin sulfate	Sigma-Aldrich	Cat#K4000
LipofectamineTM 2000 Transfection Reagent	Invitrogen	Cat#11668027
Luria-Bertani (LB) Broth (1×)	Gibco	Cat#10855001
NuPAGE™ 4× LDS sample buffer	Invitrogen	Cat#NP0007
Opti-MEM™ Reduced Serum Medium	Gibco	Cat#31985062
Pierce Protease Inhibitor XL Capsules, EDTA-free	Thermo Fisher Scientific	Cat#A37989
Potassium phosphate dibasic (K2HPO4)	Sigma-Aldrich	Cat#P8281-500G
Potassium phosphate monobasic (KH2PO4)	Sigma-Aldrich	Cat#P5379-500G
Precision Plus Protein Dual Colour Standards	Bio-Rad	Cat#1610374
SimplyBlue™ SafeStain	Thermo Fisher Scientific	Cat#LC6065
S.O.C. Medium	Thermo Fisher Scientific	Cat#15544034
Sodium chloride (NaCl)	Sigma-Aldrich	Cat#S7653-1KG
Sodium hydroxide (NaOH)	Sigma-Aldrich	Cat#1310-73-2
SUMO-Specific Protease 2 (SENP2)	Leadgene	Cat#LDG0015RG
Tris-Glycine SDS Running buffer	Thermo Fisher Scientific	Cat#LC2675
Tris-HCl	Sigma-Aldrich	Cat#T15760
Triton X-114	Sigma-Aldrich	Cat#X114
Tryptone	Sigma-Aldrich	Cat#T7293-1KG
Water for Injection (WFI) for Cell Culture	Thermo Fisher Scientific	Cat# A1287301
Yeast extract	Sigma-Aldrich	Cat#Y1625-1KG
2% uranyl acetate	Agar Scientific	Cat#AGR1260D

**Critical commercial assays**		

Pierce BCA Protein Assay Kit - Reducing Agent Compatible	Thermo Fisher Scientific	Cat#23250
Pierce High Capacity Endotoxin Removal Spin Column, 0.5 ml	Thermo Fisher Scientific	Cat#88274
Pierce LAL Chromogenic Endotoxin Quant kit	Thermo Fisher Scientific	Cat#A39553

**Experimental models: Cell lines**		

Tau RD P301S FRET Biosensor	ATCC	CRL-3275

**Recombinant DNA**		

pETM11-Sumo3-TauWT2N4R	This paper	Addgene Plasmid Cat#249347

**Software and algorithms**		

ImageJ	Schneider et al.^22^	https://imagej.nih.gov/ij/
FlowJo	Baumgarth^23^	https://www.flowjo.com/
UNICORN™ Control Software	Cytiva	https://www.cytivalifesciences.com/en/us/products/items/unicorn-61-p-03378

**Other**		

AKTA Pure 25M	Cytiva	N/A
Avanti JXN-26	Beckman Coulter	N/A
BD Fortessa X20	BD Biosciences	N/A
EmulsiFlex-C5 French Press homogeniser	Avestin	N/A
EVOS FL Auto imaging system	Termo Fisher Scientific	N/A
Heraeus Multifuge 3S-R	Thermo Fisher Scientific	N/A
Innova®44 Incubator Shaker	New Brunswick Scientific	N/A
JLA-8.1000 Fixed-Angle Aluminium rotor	Beckman Coulter	N/A
pH Meter	Mettler-Toledo	N/A
PELCO easiGlow Glow Discharge Cleaning System	PELCO	N/A
SpectraMax M5e Multimode Microplate reader	Molecular Devices	N/A
Tecnai-12 Transmission Electron Microscope	FEI	N/A
0.5 mL Protein LoBind Tube	Eppendorf	Cat#022431064
1.5 mL Protein LoBind Tube	Eppendorf	Cat#022431081
15 mL Protein LoBind Tube	Eppendorf	Cat#0030122208
50 mL Protein LoBind Tube	Eppendorf	Cat#0030122240
100 mL bottle	Thermo Fisher Scientific	Cat#10339001
250 mL Duran® bottle	Merck Millipore	Cat#Z305189
1 L Polypropylene Bottle Assembly	Beckman Coulter	Cat# C31597
0.22 μm syringe filters	Starlab	Cat# E4780-1226
0.45 μm syringe filters	Starlab	Cat# E4780-1456
Nalgene Rapid Flow Sterile Disposable Bottle Top Filters - 500 mL, 0.2 μm	Nalgene filtration products	Cat#291-4520
300-mesh carbon-coated copper grids	TAAB Laboratories	Cat# C267
Cuvettes	VWR	Cat#634-0676
HisTrap™ HP, 5 mL	Cytiva	Cat#17524802
HiTrap™ Capto™S, 5 mL	Cytiva	Cat#17544123
Novex WedgeWell 8%–16% Tris-Glycine Gel 1mmx15 wells	Invitrogen	Cat#XP08165BOX
SnakeSkin™ Dialysis Tubing 22 mm diameter, 3.5 kDa MWCO	Thermo Fisher Scientific	Cat#68035
SnakeSkin™ Dialysis Clips	Thermo Fisher Scientific	Cat#68011
Slide-A-Lyzer™ Float Buoys	Fisher Scientific	Cat#PI66430
Pierce Protein Concentrator PES, 10K MWCO, 5–20 ml	Thermo Fisher Scientific	Cat#88528

## Materials and equipment

***Note:*** Materials listed here are scaled for protein expression and purification from a 3 L bacterial culture.

***Note:*** This protocol requires access to a liquid chromatography-mass spectrometry (LC-MS) instrument or mass spectrometry core facility to verify the molecular mass and purity of the purified protein before downstream applications.

### Materials for tau expression

**Table:** Terrific Broth (TB) base

Reagent	Final concentration	Amount
Tryptone	13.3 g/L	36 g
Yeast extract	26.7 g/L	72 g
Glycerol	0.44% v/v	12 mL
ddH_2_O	N/A	Up to 2,700 mL
**Total**	**N/A**	**2700 mL**

Mix well, add 900 mL to each of three 2 L Erlenmeyer flasks, cover and autoclave. Prepare the day before use and store at RT.

**Table:** 10× TB salts stock

Reagent	Final concentration	Amount
KH_2_PO_4_	0.17 M	23.1 g
K_2_HPO_4_	0.72 M	125.4 g
ddH_2_O	N/A	Up to 1,000 mL
**Total**	**N/A**	**1,000 mL**

Mix well and autoclave. Prepare the day before use and store at RT.

**Table:** IPTG stock

Reagent	Final concentration	Amount
IPTG	100 mM	0.952 g
ddH_2_O	N/A	Up to 40 mL
**Total**	**N/A**	**40 mL**

Prepare fresh before use and keep on ice.

### Materials for tau purification

**CRITICAL:** Prepare, adjust pH, and store all stock solutions and buffers at 4°C.

**CRITICAL:** All purification buffers must be prepared fresh on the day of use to ensure stability of the reducing agents and protease inhibitors.

***Note:*** Buffers listed in this section are sterile-filtered and degassed at 4°C using a 0.2 μm bottle top filter connected to a vacuum line and attached to a sterile bottle. Please note that if degassing is performed at room temperature due to laboratory constraints, the buffers should be thoroughly cooled to 4°C prior to use.

**CRITICAL:** BME is a toxic and flammable chemical that requires handling in a fume hood. Exercise proper laboratory safety protocols.

**Table:** HEPES stock solution

Reagent	Final concentration	Amount
HEPES	1 M	238.3 g
ddH_2_O (cold)	N/A	Up to 1,000 mL
**Total**	**N/A**	**1,000 mL**

Adjust pH to 7.6 at 4°C with 10 M NaOH.

***Note:*** Prepare fresh HEPES stock for each purification round and seal well to minimize exposure to air and microorganisms.

**Table:** NaCl stock solution

Reagent	Final concentration	Amount
NaCl	2 M	116.88 g
ddH_2_O (cold)	N/A	Up to 1,000 mL
**Total**	**N/A**	**1,000 mL**

***Note:*** The stock solution is suitable for long-term storage if kept in sealed container at 4°C.

**Table:** Imidazole stock solution

Reagent	Final concentration	Amount
Imidazole	1 M	68.08 g
ddH_2_O (cold)	N/A	Up to 1,000 mL
**Total**	**N/A**	**1,000 mL**

**CRITICAL:** Imidazole is a chemical with corrosive and reproductive hazards. Exercise proper laboratory safety protocols upon handling. The stock solution is suitable for long-term storage if kept in a sealed container at 4°C, protected from light.

**Table:** Lysis buffer

Reagent	Final concentration	Amount
1 M HEPES, pH 7.6	25 mM	12.5 mL
2 M NaCl	150 mM	37.5 mL
1 M Imidazole	10 mM	5 mL
0.5 M Bond breaker TCEP, neutral pH	1 mM	1 mL
ddH_2_O (cold)	N/A	444 mL
**Total**	**N/A**	**500 mL**
DNase I	1.68 U/mL	5 μL
100× Protease Inhibitor XL Capsules EDTA-free	1×	1 tablet

Adjust pH to 7.6 at 4°C with 37% HCl. Filter through a bottle top 0.2 μm membrane filter before use.

### Ni-NTA buffers

**Table:** HisTrap A1 buffer

Reagent	Final concentration	Amount
1 M HEPES, pH 7.6	25 mM	25 mL
2 M NaCl	150 mM	75 mL
1 M Imidazole	10 mM	10 mL
0.5 M Bond breaker TCEP, neutral pH	1 mM	2 mL
ddH_2_O (cold)	N/A	888 mL
**Total**	**N/A**	**1,000 mL**
100× Protease Inhibitor XL Capsules EDTA-free	1×	2 tablets

Adjust pH to 7.6 at 4°C with 37% HCl. Degas and filter through a bottle top 0.2 μm membrane filter at 4°C before use.

**Table:** HisTrap A2 buffer

Reagent	Final concentration	Amount
1 M HEPES, pH 7.6	25 mM	12.5 mL
2 M NaCl	150 mM	37.5 mL
1 M Imidazole	10 mM	5 mL
0.5 M Bond breaker TCEP, neutral pH	1 mM	1 mL
ddH_2_O (cold)	N/A	444 mL
**Total**	**N/A**	**500 mL**
100% Triton-X114	0.1%	500 μL

Adjust pH to 7.6 at 4°C with 37% HCl. Degas and filter through a bottle top 0.2 μm membrane filter at 4°C before use.

**CRITICAL:** Add Triton-X114 to the solution after filtration. Mix thoroughly at 4°C, using gentle stirring (e.g. a magnetic stirrer at low speed) until fully homogeneous, taking care to avoid excessive foaming or air incorporation.

**Table:** HisTrap B buffer

Reagent	Final concentration	Amount
1 M HEPES, pH 7.6	25 mM	12.5 mL
2 M NaCl	150 mM	37.5 mL
1 M Imidazole	500 mM	250 mL
0.5 M Bond breaker TCEP, neutral pH	1 mM	1 mL
ddH_2_O (cold)	N/A	199 mL
**Total**	**N/A**	**500 mL**

Adjust pH to 7.6 at 4°C with 37% HCl. Degas and filter through a bottle top 0.2 μm membrane filter at 4°C before use.

**Table:** Dialysis buffer

Reagent	Final concentration	Amount
1 M HEPES, pH 7.6	25 mM	112.5 mL
2 M NaCl	100 mM	225 mL
14.3 M BME	5 mM	1.6 mL
ddH_2_O (cold)	N/A	4,160.9 mL
**Total**	**N/A**	**4,500 mL**

Mix thoroughly and adjust pH of the dialysis buffer to 7.6 at 4°C with 10 M NaOH.

### Ni-NTA rebind buffers

**Table:** HisTrap Rebind C buffer

Reagent	Final concentration	Amount
1 M HEPES, pH 7.6	25 mM	25 mL
2 M NaCl	100 mM	50 mL
1 M Imidazole	10 mM	10 mL
ddH_2_O (cold)	N/A	915 mL
**Total**	**N/A**	**1,000 mL**
14.3 M BME	5 mM	350 μL

Adjust pH to 7.1 at 4°C with 37% HCl. Degas and filter through a bottle top 0.2 μm membrane filter at 4°C before use.

**Table:** HisTrap Rebind D buffer

Reagent	Final concentration	Amount
1 M HEPES, pH 7.6	25 mM	12.5 mL
2 M NaCl	100 mM	25 mL
1 M Imidazole	500 mM	250 mL
ddH_2_O (cold)	N/A	212.5 mL
**Total**	**N/A**	**500 mL**
14.3 M BME	5 mM	175 μL

Adjust pH to 7.1 at 4°C with 37% HCl. Degas and filter through a bottle top 0.2 μm membrane filter at 4°C before use.

### CIEX buffers

**Table:** CIEX E buffer

Reagent	Final concentration	Amount
1 M HEPES, pH 7.6	25 mM	25 mL
2 M NaCl	100 mM	50 mL
ddH_2_O (cold)	N/A	925 mL
**Total**	**N/A**	**1,000 mL**
14.3 M BME	5 mM	350 μL

Adjust pH to 7.1 at 4°C with 37% HCl. Degas and filter through a bottle top 0.2 μm membrane filter at 4°C before use.

**Table:** CIEX F buffer

Reagent	Final concentration	Amount
1 M HEPES, pH 7.6	25 mM	12.5 mL
2 M NaCl	1 M	250 mL
ddH_2_O (cold)	N/A	237.5 mL
**Total**	**N/A**	**500 mL**
14.3 M BME	5 mM	175 μL

Adjust pH to 7.1 at 4°C with 37% HCl. Degas and filter through a bottle top 0.2 μm membrane filter at 4°C before use.

### Materials for residual endotoxin removal

**CRITICAL:** Prepare all stock solutions and buffers fresh on the day of use in a biosafety cabinet to maintain endotoxin-free conditions.

***Note:*** Water for Injection (WFI) (Thermo Fisher Scientific) was used in this protocol to prepare endotoxin-free reagents for final endotoxin removal. However, alternative water sources may also be suitable, providing they are certified or validated to be endotoxin-free.

**Table:** Regeneration buffer

Reagent	Final concentration	Amount
NaOH	0.2 N	0.2 g
100% EtOH	95%	23.75 mL
WFI	N/A	1.25 mL
**Total**	**N/A**	**25 mL**

Filter through a 0.22 μm syringe filter.

**Table:** NaCl stock solution

Reagent	Final concentration	Amount
NaCl	2 M	2.922 g
WFI	N/A	25 mL
**Total**	**N/A**	**25 mL**

Filter through a 0.22 μm syringe filter.

**Table:** Endotoxin-free buffer

Reagent	Final concentration	Amount
Tris-HCl	30 mM	0.118 g
2 M NaCl	0.2 M	2.5 mL
WFI	N/A	22.5 mL
**Total**	**N/A**	**25 mL**

Filter through a 0.22 μm syringe filter. Determine pH using pH strips in a biosafety cabinet. Adjust pH to 6–8 with sterile-filtered 0.1 M NaOH.

### Materials for heparin-induced *in vitro* tau aggregation

**CRITICAL:** Prepare the stock solution fresh on the day of use in a biosafety cabinet to maintain endotoxin-free conditions.

**Table:** Heparin stock solution

Reagent	Final concentration	Amount
Heparin	700 μM	12.6 mg
WFI	N/A	1 mL
**Total**	**N/A**	**1 mL**

Filter through a 0.22 μm syringe filter.

**CRITICAL:** Heparin molecular weight can vary from batch to batch, normally falling between 15–19 kDa. Check the molecular weight and adjust the amount of heparin needed accordingly for a final concentration of 700 μM. In this specific example, heparin molecular weight was 18 kDa.

## Step-by-step method details

### Human recombinant WT 2N4R tau expression

**Timing: 3 days**

This step transforms the kanamycin-resistant expression plasmid pETM11-Sumo3-TauWT2N4R (Addgene) into the chemically competent BL21 (DE3) *E. coli*. Bacterial expression of human recombinant WT 2N4R tau protein is then induced with isopropyl-β-D-thiogalactopyranoside (IPTG). Cell pellets are collected for further purification.

1. Transform BL21 (DE3) Competent Cells with pETM11-Sumo3-TauWT2N4R vector:
  a. Thaw BL21 (DE3) Competent Cells for 15 min on ice.
  b. Add 15 ng of the pETM11-Sumo3-TauWT2N4R plasmid directly into 50 μl of the bacterial suspension. Mix the suspension by gently flicking the tube.
    **CRITICAL:** Do not vortex.
  c. Incubate the cells on ice for 30 min.
  d. Heat-shock at 42°C for 30 s, then immediately after, transfer the cells directly on ice for 5 min.
  e. Add 450 μL of S.O.C. medium at room temperature (RT) and incubate for 1 h at 37°C with orbital shaking at 180 rpm.
    ***Note:*** Unless otherwise stated, bacterial cultures were incubated with shaking in an Innova®44 Incubator Shaker, and all reported shaking speeds (rpm) correspond to this instrument.
  f. Pre-warm LB agar plates containing 50 μg/mL kanamycin.
  g. Spread 50 μL of the bacterial suspension on the agar plate using a sterile cell spreader.
  h. Incubate at 37°C for 14–16 h.
    **Pause point:** After transformation, the agar plate can be stored at 4°C. For optimal protein expression, we recommend using freshly transformed colonies.
2. Inoculate a single colony of the transformed bacteria into 40 mL Luria-Bertani (LB) broth supplemented with 50 μg/mL kanamycin, in a 250 mL Erlenmeyer flask. Repeat 3× times to prepare a total of three starter cultures.

***Alternatives:*** A glycerol stock of pETM11-Sumo3-TauWT2N4R-transformed BL21 (DE3) bacteria can be used to inoculate the starter cultures. To recover bacteria from the glycerol stock, use a sterile loop or a pipette tip to gently scrape the still frozen stock and inoculate LB broth as explained in step 2.

3. Incubate the starter cultures at 37°C for 14–16 h in an orbital shaker with 180 rpm agitation.

***Note:*** A bacterial glycerol stock can be prepared at the end of this incubation step. Gently mix 500 μl of bacterial culture with 500 μl of autoclaved 50% glycerol in a cryovial and store at −80 °C for up to five years.

4. Transfer each starter culture into a corresponding 2 L Erlenmeyer flask filled with 900 mL of autoclaved Terrific Broth (TB) medium, supplemented with 100 mL of 10× TB salts and 50 μg/mL kanamycin.

***Note:*** For improved aeration, cultures may be grown at lower fill volumes (e.g., 400–500 mL in a 2 L flask), although the conditions described here consistently yielded sufficient recombinant tau for downstream purification.

5. Grow cells at 37°C with 200 rpm agitation.
6. Measure the optical density at 600 nm (OD_600_) every 30 min by transferring 1 mL of bacterial culture to a cuvette. When the OD_600_ reaches a value of 0.5–0.7 (2–3 h), induce protein expression by adding 5 mL of 100 mM IPTG (final concentration of 0.5 mM).

***Note:*** OD_600_ was determined here from absorbance at 600 nm measured with a SpectraMax M5e Multimode Microplate Reader, equipped with a cuvette chamber.

7. Incubate cells first for 1 h at 37°C with 225 rpm agitation, then for further 14–16 h at 25°C with 200 rpm agitation.
8. Transfer the cultures to three 1 L Polypropylene Bottles. Harvest cell pellets by centrifuging for 30 min at 5,000 × *g* and 4°C using the Avanti JXN-26 centrifuge with JLA-8.1000 fixed-angle aluminum rotor or equivalent.

**Pause point:** If unable to proceed with the centrifugation at the required timepoint, it is possible to store cells on ice for a few hours to stop the bacterial growth and prevent cell death. Do not freeze the cultures at this point.

9. Discard the supernatant and resuspend pellets in 25 mL of freshly prepared, ice-cold lysis buffer per 1 L of pelleted culture.

**CRITICAL:** Protease inhibitors are added to the lysis buffer to minimize protein degradation by host proteases during bacterial lysis in the next step**.**

**CRITICAL:** Keep the cells on ice for resuspension, to minimise proteolytic degradation and preserve protein integrity.

**CRITICAL:** Ensure thorough resuspension until no clumps are visible to prevent clogging of the homogenizer and sample loss in the next steps.

**Pause point:** Following resuspension in ice-cold lysis buffer, the intact bacterial cells can be stored at −80°C for up to four weeks before proceeding with bacterial lysis. Alternatively, samples may be stored after clarification (Step 14), but only one of these pause points is recommended to avoid multiple freeze-thaw cycles.

### Bacterial lysis

**Timing: 4–5 h**

Bacteria expressing 2N4R WT tau are lysed in this step to release the intracellular tau. We exploit the unique biophysical properties of tau by combining the French Press homogenization conventionally used for large-scale bacterial lysis with direct boiling method. This approach enhances the removal of impurities and minimizes tau degradation by denaturing bacterial proteases while efficiently extracting the thermostable tau protein.

10. Thaw bacteria suspension on ice.

***Alternatives:*** Place cells in an unheated water bath containing water at ambient laboratory RT for faster thawing time.

11. Lyse the cells using EmulsiFlex-C5 French Press homogeniser operating at 15 kpsi. Approximately 4 to 5 cycles are needed to completely disrupt the cells.

***Note:*** Keep the lysate at a low temperature with 4°C cold circulation water.

***Alternatives:*** Sonication can be used to lyse the cells in the event of small-scale purification.

12. Transfer lysates to 50 mL Protein LoBind tubes on ice.
13. Centrifuge for 1 h at 4,500 × *g* at 4°C using the Heraeus Multifuge 3SR centrifuge or equivalent to remove cell debris and insoluble fractions.
14. Transfer supernatants to clean 50 mL Protein LoBind tubes and discard pellets.

**Pause point:** Supernatant can be stored up to one week at −80°C before continuing with direct boiling in the next step. If the bacterial cells were previously stored at −80°C following Step 9, we recommend skipping this pause point and continuing directly with the protocol to avoid multiple freeze-thaw cycles.

15. Incubate the supernatants in 70°C water bath for 10 min, then allow them to cool at RT for 10–15 min before centrifugation.

***Note:*** Loosen the lids a little before the incubation to prevent pressure build-up during heating.

***Note:*** Ensure falcons are maximally submerged while preventing the water line from reaching the lid.

16. Centrifuge again for 1 h at 4,500 × *g* at 4°C using the Heraeus Multifuge 3SR centrifuge or equivalent.
17. Filter supernatants through 0.45 μm syringe filter and pool to a sterile laboratory plastic bottle on ice. Continue with immobilized metal affinity chromatography (IMAC) in the next step.

***Note:*** Keep 20 μL aliquot of the clarified cell lysate at 4°C for SDS-PAGE analysis.

### Endotoxin-free human recombinant WT 2N4R tau purification

**Timing: 2 days**

This step describes the purification of endotoxin-free, human recombinant WT 2N4R tau protein. The endotoxin removal is achieved in the first step by a combination of affinity chromatography with on-column Triton X-114 detergent wash at 4°C. The protocol builds on one of the oldest and most effective methods for removal of endotoxin from purified protein and plasmid DNA, based on the Triton X-114 property to undergo phase separation into aqueous and hydrophobic phase.^24^^,^^25^^,^^26^ It is cheap, efficient, reproducible, and ensures maximum protein recovery. The second purification step, cation exchange chromatography, increases tau purity while further reducing endotoxin contamination.

**CRITICAL:** All purification steps are carried out at 4°C. Ensure all buffers are fresh, sterile-filtered, degassed, and at 4°C before use.

**Figure 1:**
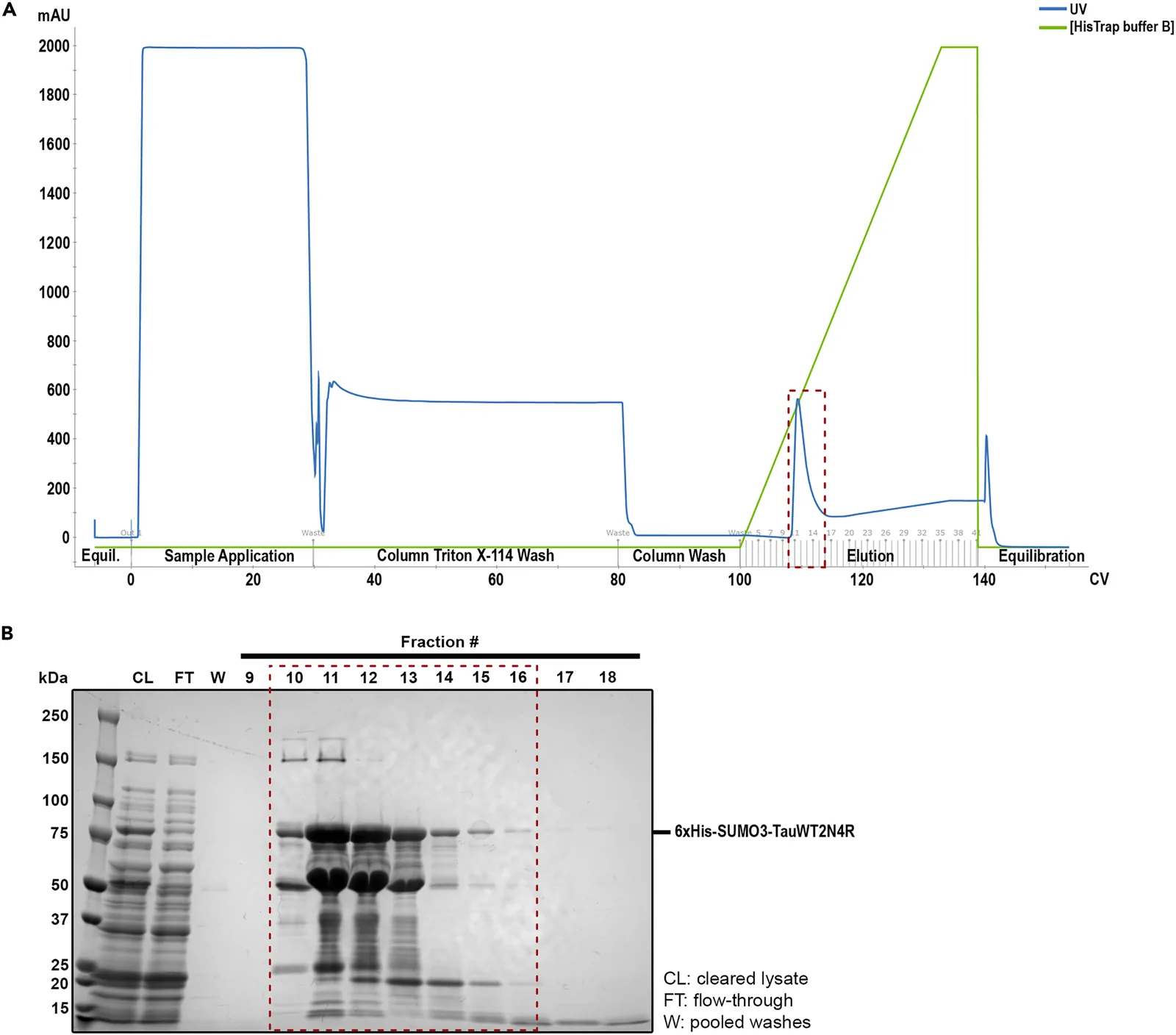
Purification of recombinant 6xHis-SUMO3-TauWT2N4R by IMAC (A) Chromatogram showing the elution profile of recombinant 6xHis-SUMO3-TauWT2N4R from a HisTrap HP column during immobilized metal affinity chromatography (IMAC). (B) SDS-PAGE analysis and SimplyBlue SafeStain visualization of collected fractions. Recombinant 6xHis-SUMO3-TauWT2N4R (∼75 kDa) elutes predominantly in fractions 10–16. Fractions pooled for subsequent purification steps are highlighted in red.

***Note:*** Prepare samples for SDS-PAGE by mixing 5 μL of sample with 6.25 μL of 4× loading buffer and 13.75 μL of ddH_2_O. Boil at 70°C for 10 min before loading onto the gel.

***Note:*** To dramatically shorten the gel staining and destaining times, follow the manufacturer’s protocol with the following modifications: loosely cover the staining container and heat in a microwave oven at full power for 20–30 s. Do not allow the solution to boil. Gently shake the gel at RT on an orbital shaker for 15 min. Decant the stain and rinse the gel once with ddH_2_O.

**Figure 2:**
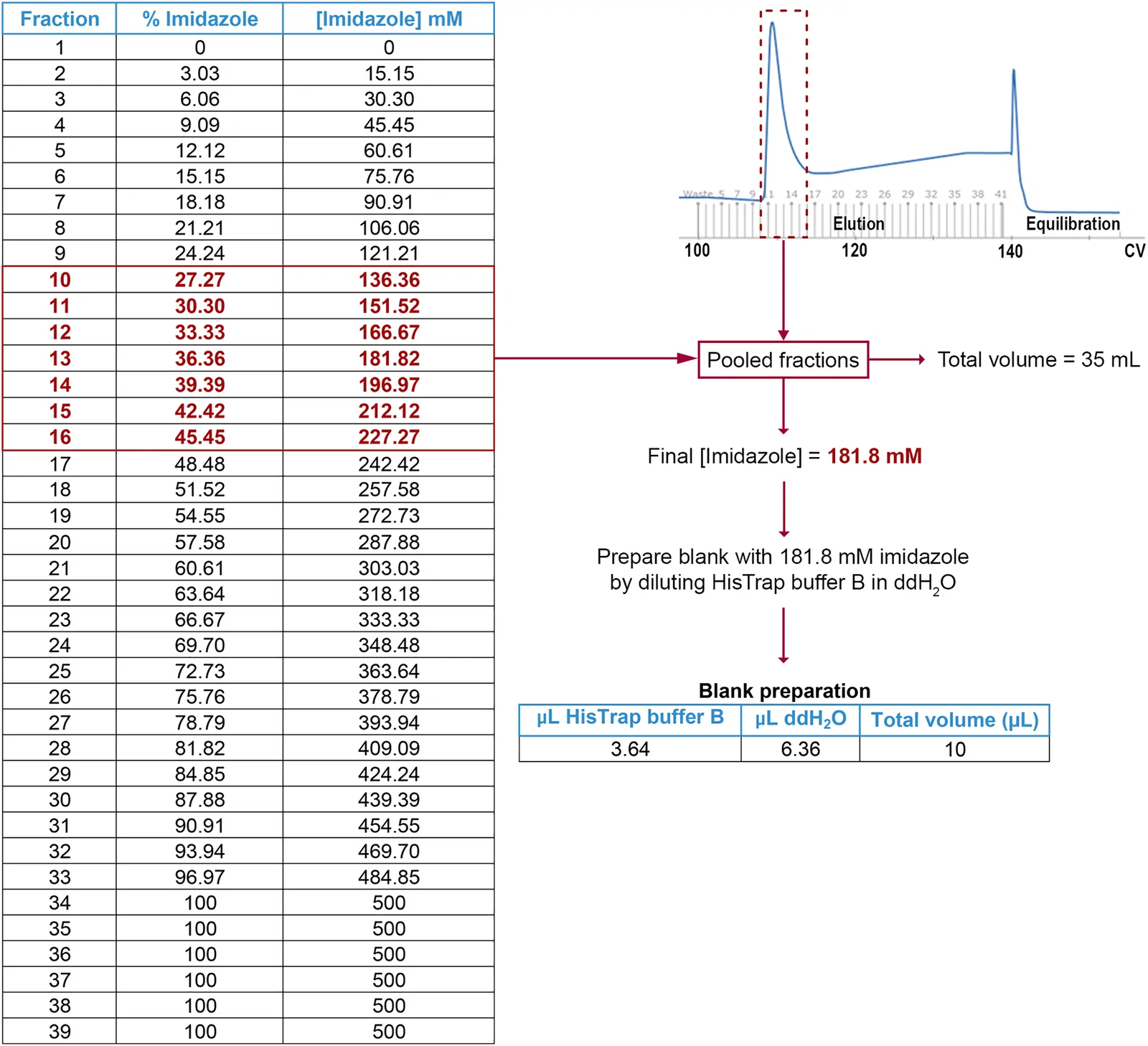
Determination of imidazole concentration in pooled IMAC fractions Table showing the percentage and calculated concentration of imidazole present in individual IMAC elution fractions. To determine protein concentration in pooled fractions by NanoDrop, first calculate the imidazole concentration by averaging the imidazole concentration across the pooled fractions. Prepare a matching NanoDrop blank by diluting HisTrap buffer B (500 mM imidazole) in ddH_2_O to the equivalent final imidazole concentration of the pooled sample (181.8 mM).
  b. Prepare a blank solution by diluting HisTrap Buffer B with ddH_2_O to achieve an imidazole concentration equivalent to that of the pooled fractions (Figure 2).
  c. Nanodrop can then be used to roughly evaluate protein concentration.

***Note:*** Expected yield is ∼ 1–1.1 mg/mL.

18. Perform an IMAC using the ÄKTA Pure 25M chromatography system or equivalent in combination with a 5 mL HisTrap HP column. Use 5 mL/min flow-rate for all steps of the IMAC.
  a. Wash the column with 5 column volumes (CVs) of double distilled water (ddH_2_O).
  b. Equilibrate the column with 5 CVs of HisTrap buffer A1.
  c. Apply sample onto the column using sample pump (∼100–150 mL from 3 L bacterial culture).
    ***Note:*** Inject fixed sample volume unless the ÄKTA system is equipped with an air sensor to detect air during sample application.
  d. Collect a sample of the flow-through (20 μL) for SDS-PAGE analysis.
  e. Wash the column with no less than 50 CVs of HisTrap buffer A2 containing 0.1% Triton X-114.
    **CRITICAL:** This step is crucial for endotoxin removal from the tau preparation.
  f. Wash the column again with 20 CVs of HisTrap buffer A1 to remove any residual detergent and collect a sample of the wash (20 μL) for SDS-PAGE analysis.
  g. Elute 6xHis-SUMO3-tagged tau from the column using 0%–100% linear gradient of HisTrap buffer B over 33 CVs, followed by 6 CVs of 100% HisTrap buffer B. Collect 5 mL fractions in 15 mL Protein LoBind tubes during elution.
  h. Clean the HisTrap column with 15 CVs of ddH_2_O, then apply 5 CVs of 20% v/v ethanol for storage.
    ***Note:*** The column can be re-used multiple times for the same purification step of the same protein. Further cleaning with 60 CVs of 1M NaOH followed by 10 CVs of ddH_2_O is recommended after every 2 to 3 runs to remove precipitated and hydrophobic contaminants including any residual endotoxin.
19. Analyze peak fractions of the IMAC along with the clarified lysate, flow-through, and wash samples using SDS-PAGE (8%–16% Tris-Glycine gel) in combination with SimplyBlue SafeStain gel staining (Figure 1).

20. Following the SDS-PAGE analysis, pool fractions containing eluted tau (∼75 kDa) (Figure 1) to a sterile 50 mL Protein LoBind tube on ice and mix gently. Keep 20 μL aliquot for further analysis.
21. Determine protein concentration in the pooled fractions:
  a. Calculate imidazole concentration in the pooled fractions (Figure 2).

***Alternatives:*** A Bradford assay (Thermo Fisher Scientific, Cat# 23200) may be used instead of Nanodrop spectrophotometry.

**Figure 3:**
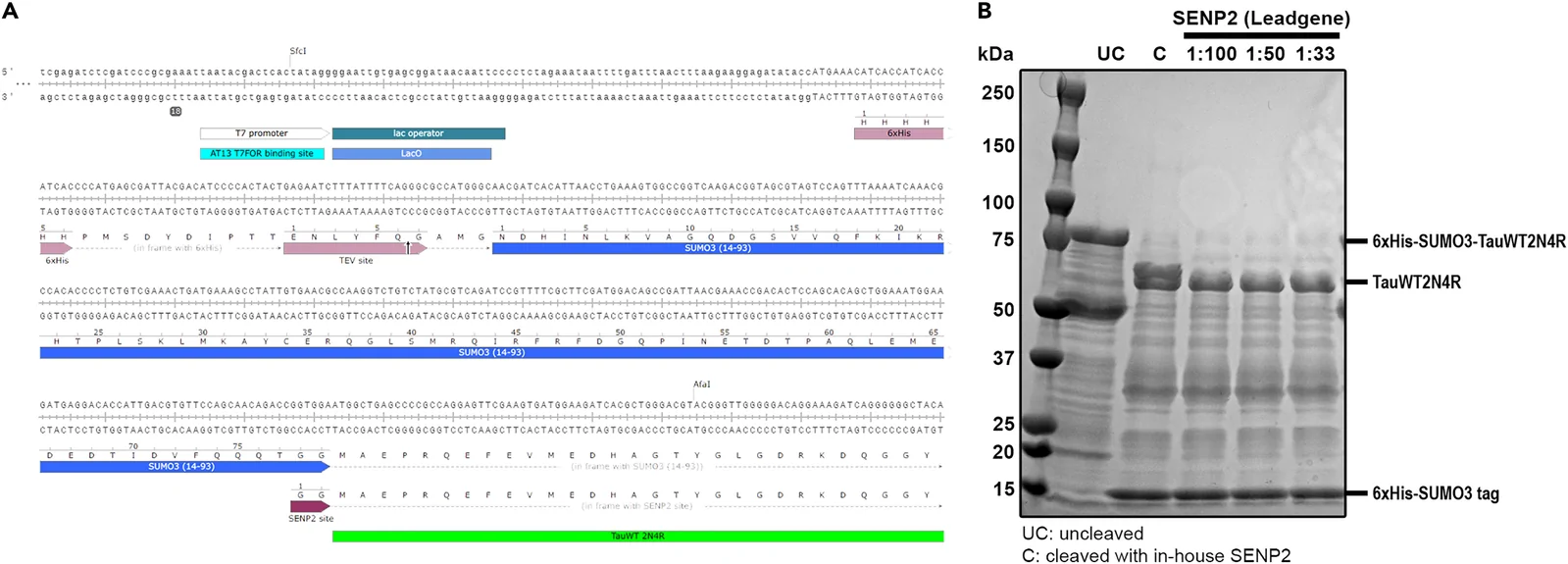
Proteolytic cleavage of 6xHis-SUMO3-tagged WT 2N4R tau (A) Schematic representation of the pETM11-SUMO3-TauWT2N4R construct showing the SENP2 protease cleavage site between the 6xHis-SUMO3 tag and tau sequence. (B) SDS-PAGE analysis comparing cleavage efficiency of in-house purified and commercially available SENP2 protease. In-house SENP2 was used at an enzyme-to-substrate ratio of 1:100 (w/w), while commercial SENP2 was tested at 1:100, 1:50, and 1:33 ratios.

***Note:*** According to manufacturer instructions, SENP2 efficiently cleaves SUMO-tagged proteins at an enzyme-to-substrate ratio of 1:50 (w/w) during an overnight incubation at 4°C under appropriate buffer conditions. Here, we tested cleavage efficiency at 1:100, 1:50 and 1:33 SENP2:protein (w/w) ratio and found that tau was efficiently cleaved at 1:100 ratio (Figure 3).

22. Add SENP2 protease (Leadgene) to the elution fractions at 1:100 protease:tau (w/w) ratio to cleave 6xHis-SUMO3 tag (Figure 3). Mix gently by pipetting up and down several times on ice.

23. Perform tag cleavage in combination with overnight dialysis at 4°C:
  a. Cut dialysis tubing (SnakeSkin) to an appropriate length (∼1 cm per mL + little extra for clips on each end) and soak in ddH_2_O for 10–15 min.
  b. Secure one end of the tubing with a dialysis clip.
  c. Load the eluted tau mixed with SENP2 protease into the tubing and seal the top end with a clip, leaving about 0.5 cm of headspace.
  d. Place the sealed tubing in 4.5 L of freshly prepared dialysis buffer (∼100-times the sample volume).
    **CRITICAL:** To avoid the magnetic stirrer from damaging the dialysis tubing, attach a float buoy to one end of the dialysis tubing and ensure it remains suspended.
  e. Cover with an aluminum foil and incubate with gentle stirring overnight at 4°C.
    ***Note:*** Dialysis enables the diffusion of small molecules, such as imidazole, into the dialysis buffer while retaining the eluted and tagless tau protein within the tubing.
    ***Alternatives:*** A Slide-A-Lyzer G3 dialysis cassette (Thermo Fisher Scientific, Cat# A52969) can be used as an alternative to the Snakeskin dialysis tubing.
24. Remove tubing from the dialysis buffer and transfer the sample to a clean Protein LoBind tube on ice. Keep 20 μL aliquot for SDS-PAGE analysis.
25. To remove uncleaved tau protein and the remaining SENP2 protease, perform an IMAC again as described in step 18 with the following modifications:
  a. Use a separate HisTrap HP column dedicated for the rebind step.
  b. Wash the column with 5 CVs of ddH_2_O.
  c. Equilibrate the column with 5 CVs of HisTrap Rebind buffer C.
  d. Apply sample onto the column using sample pump (∼35 mL).
    ***Note:*** Inject fixed sample volume unless the ÄKTA system is equipped with an air sensor to detect air during sample application.
  e. Collect the flow-through in a clean 50 mL Protein LoBind tube and keep a sample (20 μL) for SDS-PAGE analysis.
  f. Wash the column with 10 CVs of HisTrap Rebind buffer C. Collect fractions in 15 mL Protein LoBind tubes.
  g. Elute cleaved tau from the column using 0%–20% linear gradient of HisTrap Rebind buffer D over 30 CVs, followed by 5 CVs of 45% buffer D, then 5 CVs of 100% buffer D. Collect 5 mL fractions in 15 mL Protein LoBind tubes.
    ***Note:*** Cleaved protein products are typically expected to elute in the flow-through of IMAC Rebind step. However, SDS-PAGE analysis has consistently shown that in practice, some cleaved tau remains associated with the HisTrap column matrix and requires re-elution with an imidazole gradient. This behaviour likely results from non-specific interactions between the protein with the HisTrap column resin, driven by the relatively low salt concentration of the buffers and the fact that tau is an intrinsically disordered protein, which can influence its chromatographic behavior.^27^^,^^28^
  h. Clean the column with 15 CVs of ddH_2_O, then apply 5 CVs of 20% v/v ethanol for storage.
26. Analyze peak fractions, the flow-through, and wash samples, as well as the before and after overnight dialysis and cleavage samples from steps 20 and 24, as described in step 19.

***Note:*** Prepare the peak fraction, flow-through, and wash samples for gel electrophoresis by mixing 10 μL of sample with 6.25 μL of 4× loading buffer and 8.75 μL of ddH_2_O. In parallel, prepare the before and after overnight dialysis and cleavage samples from steps 20 and 24 by mixing 5 μL of sample with 6.25 μL of 4× loading buffer and 13.75 μL of ddH_2_O. Boil all samples at 70°C for 10 min before loading onto the gel.

27. Pool fractions containing eluted, cleaved tau (∼60 kDa) (Figure 4) to a sterile 100 mL bottle on ice and mix gently.

**Figure 4:**
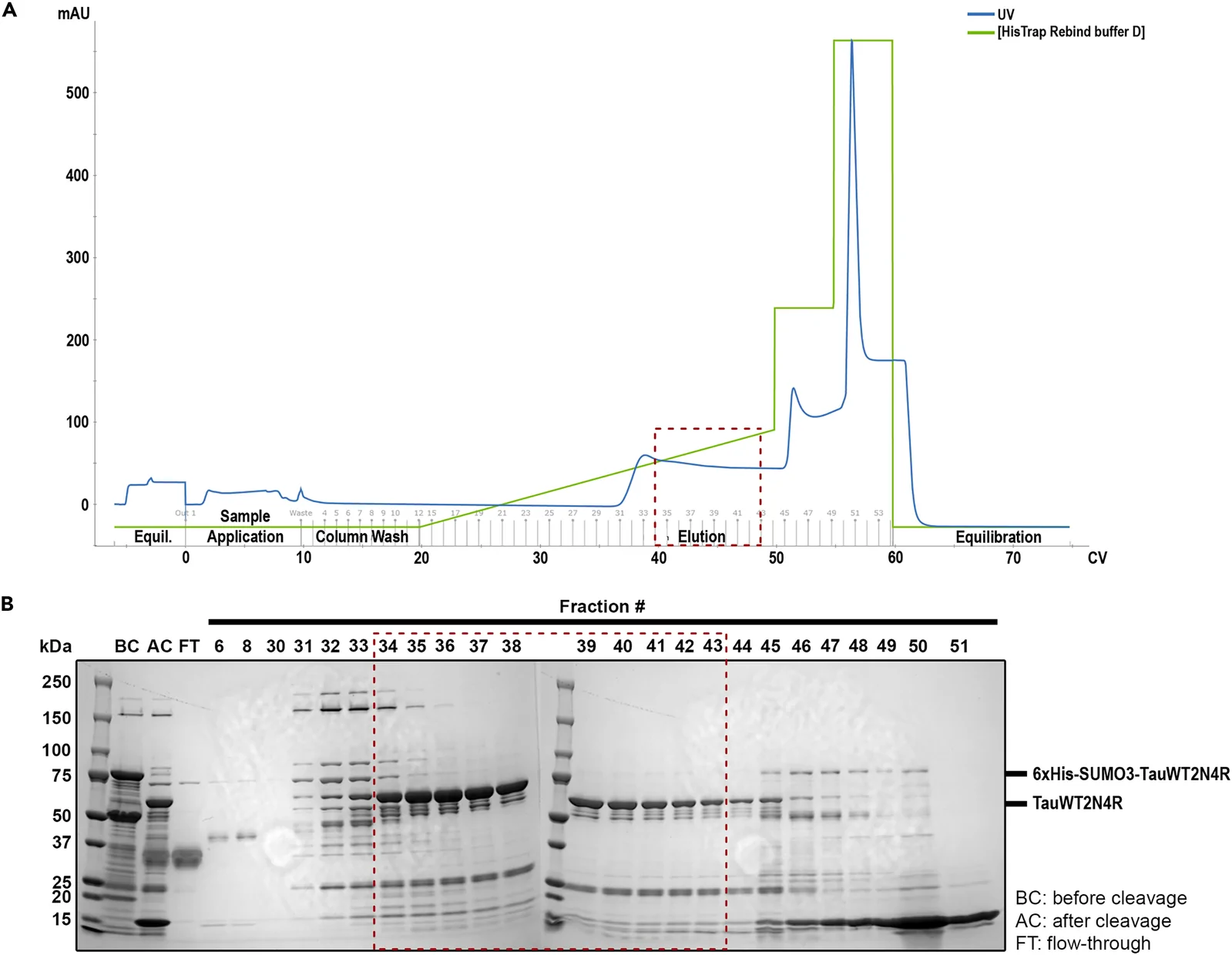
Purification of recombinant WT 2N4R Tau by IMACrebind chromatography (A) Chromatogram showing the elution profile of recombinant WT 2N4R Tau from a HisTrap HP column following SENP2-mediated cleavage of the 6xHis-SUMO3 tag. Fractions selected for further purification are highlighted in red. (B) SDS-PAGE analysis followed by Coomassie staining of eluted fractions. Fractions 34–43 contain cleaved WT 2N4R tau (∼55 kDa) with no detectable uncleaved 6xHis-SUMO3-TauWT2N4R remaining in the preparation.
28. Dilute the pooled sample with CIEX buffer E to dilute imidazole concentration and aid sample binding to the CIEX column in the following step (Figure 5).

**Figure 5:**
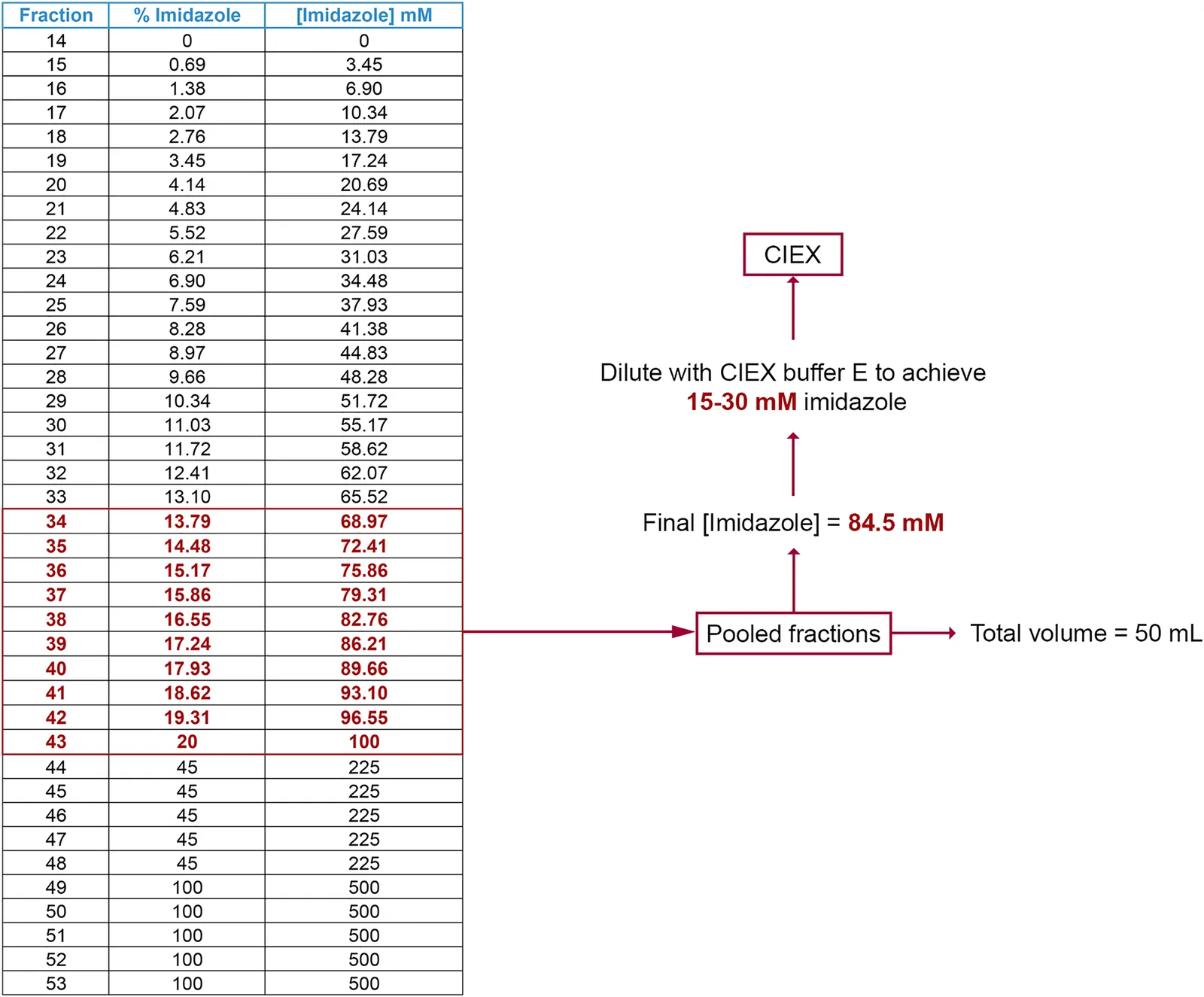
Sample preparation for CIEX Table showing the percentage and calculated concentration of imidazole present in individual IMAC elution fractions. The final imidazole concentration is determined as the mean concentration across pooled fractions. Prior to loading onto the CIEX column, the sample is diluted with CIEX buffer E to reduce the imidazole concentration to 15–30 mM and promote efficient tau binding to the resin.

**CRITICAL:** Decreasing the imidazole concentration in this step is essential for efficient binding of recombinant tau to the CIEX column. However, achieving this through higher dilution would considerably increase the sample application time in the subsequent step. We found that an imidazole concentration between 15–30 mM provides a good balance, yielding optimal recovery.

29. Perform cation exchange chromatography (CIEX) to further increase tau purity, using 5 mL HiTrap Capto S column. Use 5 mL/min flow-rate for all steps of the CIEX.
  a. Wash the column with 5 CVs of ddH_2_O.
  b. Equilibrate the column with 5 CVs of CIEX buffer E.
  c. Apply the sample onto the column using sample pump.
    ***Note:*** Inject fixed sample volume unless the ÄKTA system is equipped with an air sensor to detect air during sample application.
  d. Collect the flow-through in a sterile 250 mL Duran bottle and keep a sample (20 μL) for SDS-PAGE analysis.
  e. Wash the column with 6 CVs of CIEX buffer E. Collect fractions in 15 mL Protein LoBind tubes.
  f. Elute cleaved tau from the column using 0%–100% linear gradient of CIEX buffer F over 30 CVs, followed by 5 CVs of 100% buffer D. Collect 5 mL fractions in 15 mL Protein LoBind tubes during elution.
  g. Clean the HiTrap CaptoS column with 15 CVs of ddH_2_O, then apply 5 CVs of 20% v/v ethanol for storage.
    ***Note:*** The column can be reused multiple times for the same purification step of the same protein. Further cleaning with 2 CVs of 2 M NaCl, 4 CVs of 1 M NaOH and 2 CVs of 2 M NaCl, followed by 10 CVs of ddH_2_O is recommended after every 2 to 3 runs to remove precipitated and hydrophobic contaminants, including any residual endotoxin.
30. Analyze peak fractions, as well as the flow-through, and wash samples, as described in step 19.

***Note:*** Prepare samples by mixing 15 μL with 6.25 μL of 4× loading buffer and 3.75 μL of ddH_2_O. Boil at 70°C for 10 min before loading onto the gel.

**Figure 6:**
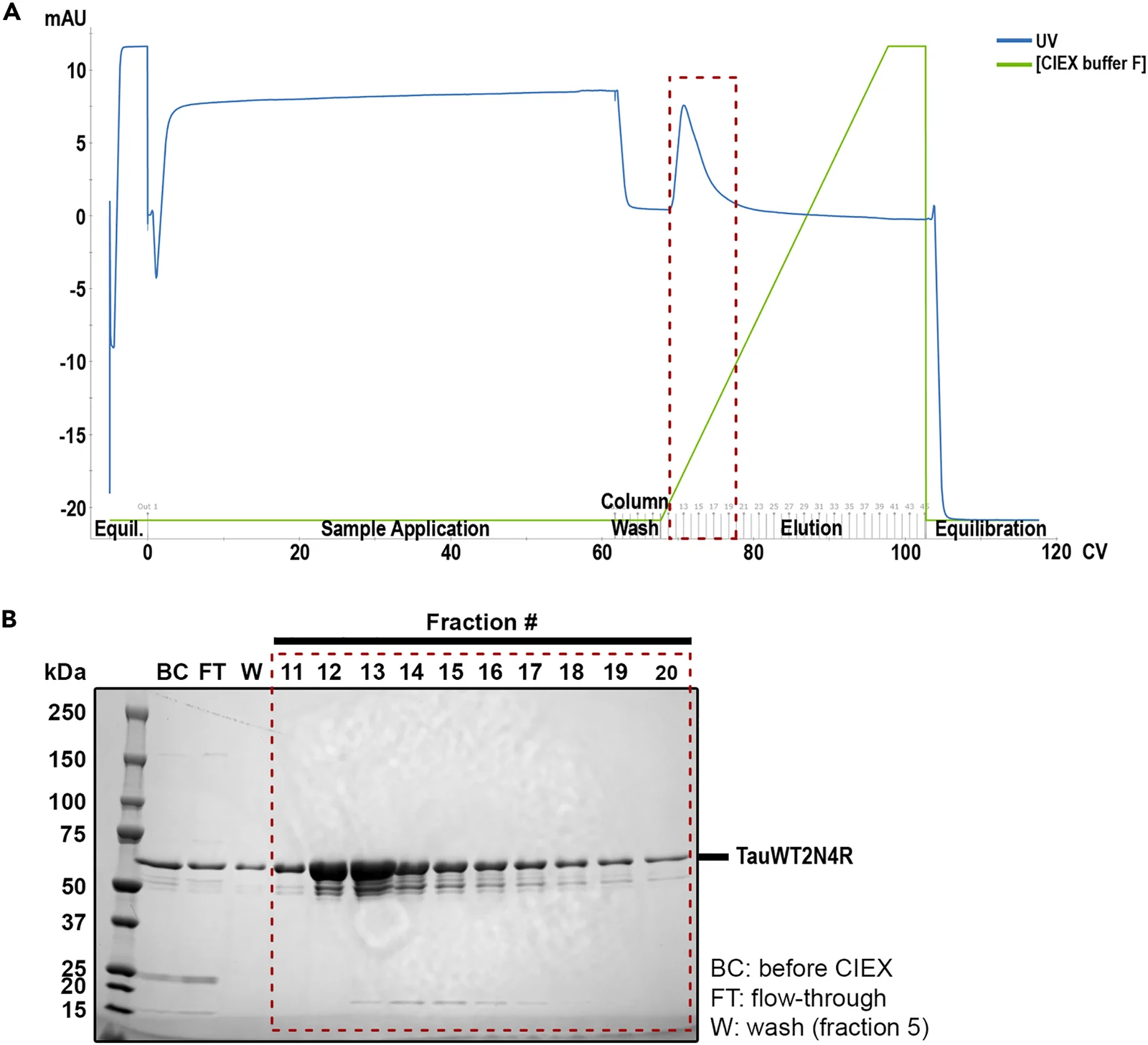
Purification of recombinant WT 2N4R Tau by CIEX (A) Chromatogram showing the elution profile of recombinant WT 2N4R tau from the CIEX column. Fractions containing recombinant tau monomer are highlighted in red. (B) SDS-PAGE analysis followed by Coomassie staining of eluted fractions.

31. Pool tau-containing fractions (∼60 kDa) (Figure 6) to a clean 50 mL Protein LoBind tube on ice. Mix gently by pipetting and keep a 20 μL sample for SDS-PAGE analysis.

**Pause point:** Store the pooled fractions at 4°C overnight.

### Tau concentration, residual endotoxin removal, and quality control

**Timing: 1–2 days**

In this step, purified tau is concentrated and incubated with an endotoxin removal resin to eliminate any residual endotoxin present in the sample. This process is necessary to ensure that the endotoxin levels in the preparation are negligible. Subsequently, a quality control assessment is performed, including determination of protein concentration, measurement of endotoxin levels, and verification of molecular mass by liquid-chromatography-mass spectrometry (LC-MS).

32. Concentrate the protein to a final volume of 2 to 3 mL using 10K MWCO Pierce Protein Concentrator PES units with 5–20 mL capacity at 4,000 × *g* and 4 °C in a benchtop centrifuge.

**CRITICAL:** Avoid over-concentrating the sample, as tau can aggregate or adhere to the membrane when the volume becomes too low. Monitor the retentate volume periodically throughout the process.

***Note:*** Concentration to the required volume typically requires 30–45 min.

***Note:*** Divide the 50 mL sample evenly among three concentrators to ensure efficient processing.

33. Transfer the concentrated protein to a sterile 15 mL Protein LoBind tube on ice.
34. Rinse the concentrator membranes with 0.5–1 mL of the eluent. Use the same rinse volume sequentially to wash all concentrators and combine the washes with the main sample to obtain a total volume of approximately 2–2.5 mL.
35. Use Pierce™ High-Capacity Endotoxin Removal Spin Columns (0.5 mL column size; Thermo Fisher Scientific) and solutions listed in the Materials for residual endotoxin removal tables of the materials and equipment section to remove residual endotoxin from the purified tau protein. Follow the manufacturer’s guidelines with minor modifications:
  **CRITICAL:** Perform all steps in a biosafety cabinet to maintain endotoxin-free conditions.
  a. Regenerate the column resin using 3.5 mL of 0.2 N NaOH in 95% ethanol for 2 h at RT on a roller with gentle end-to-end mixing.
    ***Note:*** The manufacturer recommends regeneration with either 0.2 N NaOH overnight or 0.2 N NaOH in 95% ethanol for 1–2 h. We used the latter approach to reduce regeneration time while maintaining effective column performance.
  b. Incubate the concentrated tau preparation with the column resin for 2 h at RT on a roller with gentle end-to-end mixing.
    ***Note:*** This modification extends the manufacturer’s recommended incubation time to enhance endotoxin removal.
  c. Place the column in a sterile 15 mL Protein LoBind tube and centrifuge at 500 × *g* for 1 min to collect the sample.
    ***Note:*** This modification specifies collection into a sterile Protein LoBind tube to minimize non-specific protein binding and maintain endotoxin-free conditions.
    ***Note:*** The final volume of eluted tau is ∼2.5 mL.
36. Sterile-filter the eluted sample in a biosafety cabinet using a 0.22 μm syringe filter.

**Pause point:** Store the final tau preparation at 4°C for up to 1 day.

37. Determine protein concentration using the Pierce BCA Protein Assay Reducing Agent Compatible Kit.

**Table 1:** The concentration and endotoxin levels in final tau preparation from 3 and 6 L bacterial cultures

Bacterial culture size (L)	Total tau yield (mg)	Tau concentration (mg/mL)	Endotoxin levels (EU/mL)	Endotoxin levels per mg tau (EU/mg)	Endotoxin levels in 1 ug/mL working concentration (EU/mL)
3	3.55	1.6	0.61	0.38	<0.01
6	4	1.6	2.31	1.43	<0.01

Values are representative of individual purification runs. Protein yield may vary between preparations depending on expression efficiency and recovery during downstream purification.

***Note:*** The total expected yield from a 3 L bacterial culture is ∼3.5 mg (Table 1).

38. Measure endotoxin levels in the tau preparation with Pierce Chromogenic Endotoxin Quant Kit, following manufacturer’s instructions.

***Note:*** Prepare endotoxin low standards (0.01–0.1 EU/mL).

***Note:*** Measure tau sample in triplicate, diluting it 25-fold with water for injections (WFI) to ensure the readings fall withing the linear range of the standard curve.

***Note:*** Endotoxin levels in the final tau preparation intended for incubation with TLR4-expressing immune-competent cells such as iPSC-derived microglia should be ≤ 0.01 EU/mL (Table 1).

39. Aliquot the protein into sterile 1.5 mL Protein LoBind tubes.

**Figure 7:**
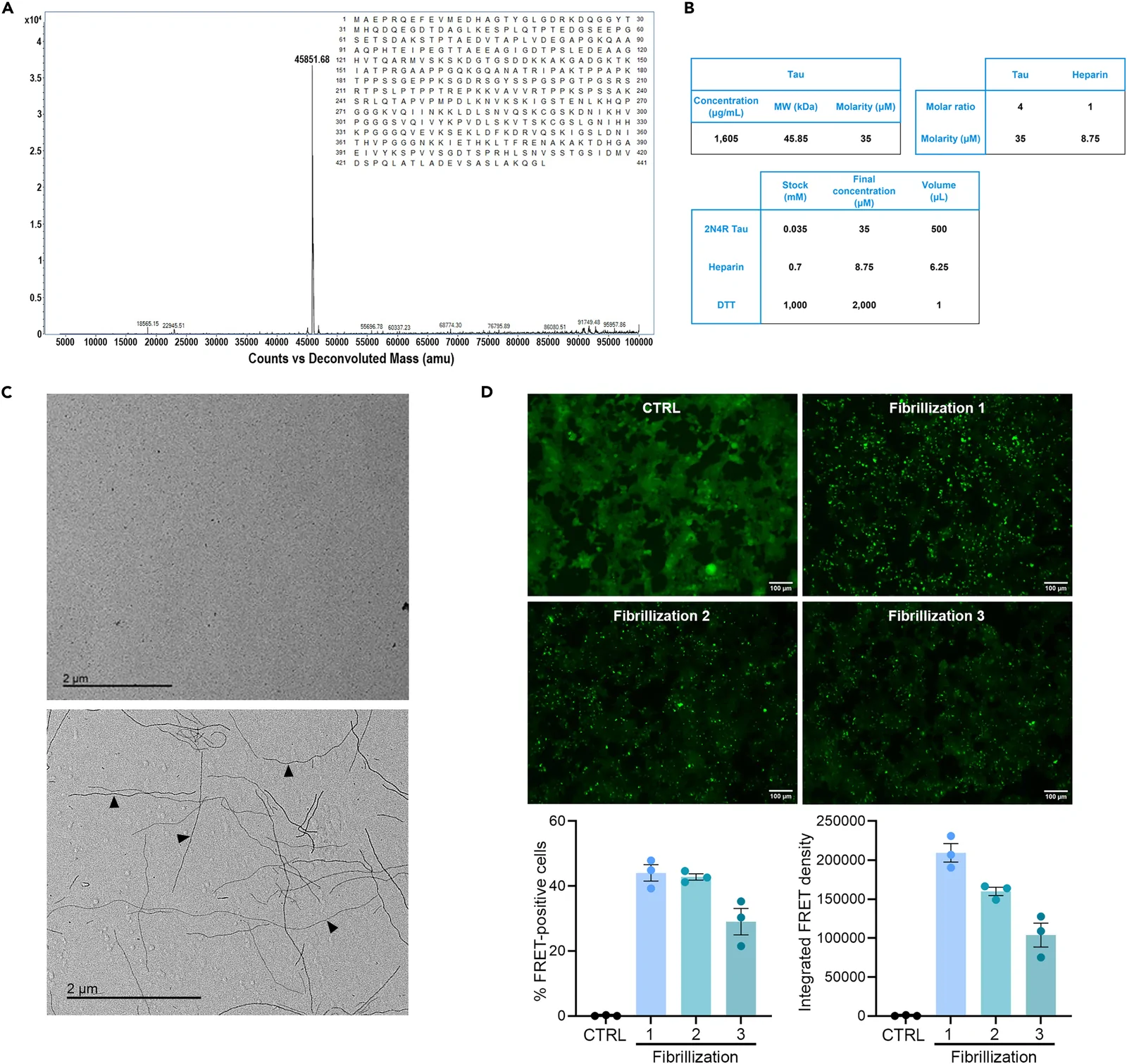
Quality control of recombinant WT 2N4R Tau monomer and heparin-induced tau fibrils (A) Verification of recombinant tau molecular mass by LC-MS. (B) Schematic showing calculation of the 4:1 tau-to-heparin molar ratio required for fibrillization, based on tau protein concentration. DTT is added to a final concentration of 2 mM. (C) Representative negative stain TEM images of recombinant tau monomer (top) and heparin-aggregated tau fibrils (bottom). Arrowheads indicate tau fibrils. Scale bar = 2 µm. (D) Representative images of tau inclusions in Tau RD P301S FRET Biosensor cells transfected with vehicle (control) or 50 nM recombinant tau fibrils (scale bar = 100 µm), together with quantification of FRET-positive cells and integrated FRET density. Data is presented as mean ± SEM, n = 3 technical replicates, one-way ANOVA, ∗∗∗∗ P < 0.0001.

***Note:*** Prepare 10 μL aliquots for protein mass verification by LC-MS (Figure 7A). Aliquot the remaining purified tau according to its intended downstream application, e.g., 500 μL aliquots for *in vitro* aggregation to fibrils, and 20 μL aliquots for functional assays in iPSC-microglia.

40. Snap-freeze in liquid nitrogen, and store at −80°C for further use.

***Note:*** In our hands, the aliquots can be stored at −80°C for up to 1 year without detectable loss of quality or performance in downstream applications.

### Heparin-induced *in vitro* tau aggregation

**Timing: 11 days**

Self-propagating activity of β-sheet rich tau aggregates is one of the hallmark pathologies in tauopathies.^29^ In this step, we induce the aggregation of purified recombinant tau monomer with polyanionic cofactor heparin at 4:1 tau:heparin molar ratio as previously described.^30^^,^^31^ The reaction mixture is kept at 37°C with constant orbital shaking for 11 days. The formation of tau fibrils is confirmed by negative-stain transmission electron microscopy (TEM) and seeding assay in Tau RD P301S FRET Biosensor.^32^^,^^33^

41. Thaw a 500 μL aliquot of purified monomeric 2N4R tau protein on ice.
42. Prepare reaction mixture for fibrillization, performing all substeps in a biosafety cabinet to maintain endotoxin-free conditions:
  a. Calculate the molarity of purified tau monomer (Figure 7B).
  b. Add appropriate volume of heparin stock to the purified tau sample to achieve a 4:1 tau:heparin molar ratio (Figure 7B).
    **CRITICAL:** Prepare heparin immediately before use to prevent degradation and sterile-filter through a 0.22 μm syringe filter to prevent contamination. Precise maintenance of the tau:heparin molar ratio is essential for consistent fibril formation.
  c. Add 1 μL of 1 M DTT to the reaction mixture to obtain a final 2 mM concentration.
  d. Mix gently by pipetting.
43. Seal the tube with parafilm and incubate at 37 °C in the Innova 44 orbital shaker set at 250 rpm for 11 days to promote tau fibril formation.

***Alternatives:*** Benchtop shaker Eppendorf Thermomixer C set at 700 rpm can also be used to promote tau aggregation.

***Note:*** The Thermomixer C speed was calculated to provide agitation equivalent to 250 rpm in the Innova 44 incubator shaker by correcting the difference in orbital diameter using the equation shown below. These conditions were validated to produce comparable fibrilization kinetics. For alternative shaking platforms, this equation can be used to estimate an equivalent shaking speed. However, validation is recommended as mechanical differences between instruments may influence aggregation kinetics.

$$r^{2}=\sqrt{r_{1}^{2}}x\frac{D_{1}}{D_{2}}$$

r_1_: speed of Innova 44 incubator shaker (250 rpm).

r_2_: speed of Eppendorf Thermomixer C.

D1: orbital diameter of Innova 44 incubator shaker (25 mm).

D2: orbital diameter of Eppendorf Thermomixer C (3 mm).

44. On day 11, remove the sample from the shaker and perform quality control (QC) analyses:
  a. Determine protein concentration using the Pierce BCA Protein Assay Reducing Agent Compatible Kit.
  b. Assess fibril formation by negative-stain TEM (Figure 7C), using the FEI Tecnai T12 transmission electron microscope operated at 120 kV or an equivalent.
    ***Note:*** To prepare samples for TEM, dilute the purified tau monomer and aggregated fibrils 1:20 in ddH_2_O. Apply 10 μL of each sample to freshly glow-discharged 300-mesh carbon-coated copper grids for 2 min. After removing excess sample with filter paper, negative-stain with 2% uranyl acetate for 20 sec, and leave to air-dry before long-term storage.
  c. Determine seeding capacity of the fibrils in Tau RD P301S FRET Biosensor system according to instructions detailed in Furman et al.^32^ with minor modifications:
    i. Plate the Tau RD P301S FRET biosensor cells at 25,000 cells/well in a 96-well flat-bottom plate.
    ii. Transfect the cells with 50 nM recombinant tau fibrils using Lipofectamine 2000 transfection reagent:
      • In a 1.5 mL Protein LoBind tube, prepare a 2× recombinant tau fibrils mix (100 nM) with Opti-MEM, ensuring that the final volume is 10 μL per well. Vortex the tube to mix.
      • In a separate tube, prepare a transfection master mix by combining 1 μL Lipofectamine 2000 with 9 μL Opti-MEM per well. Invert the tube gently to mix and incubate 5 min at RT.
      • Combine equal volumes of 2× tau fibrils mix and transfection mix, vortex, and incubate 20 min at RT to allow transfection complexes to form. This yields transfection complexes containing 50 nM recombinant tau fibrils.
      • Add 20 μL of transfection complexes per well without removing any medium.
      ***Note:*** Prepare a negative control by mixing 10 μL transfection mix with 10 μL Opti-MEM per well.
      ***Note:*** Prepare sufficient tau fibrils and transfection mixes for three technical replicates per condition, including the negative control, and account for pipetting losses.
    iii. 48 h after transfection, check for the presence of tau inclusions in the biosensor cells (Figure 7D). Acquire representative images using an EVOS FL Auto imaging system, or equivalent, and visualize them in ImageJ.
    iv. Measure CFP and FRET signals using a 405 nm excitation laser on the BD Fortessa ×20 flow cytometer, with 405/50 nm and 525/50 nm band-pass emission filters, respectively.
      ***Note:*** The 488 nm laser is switched off during FRET measurement so that the fluorescence detected by the 525/50 nm band-pass filter originates only from energy transfer from CFP to YFP, instead of direct excitation of YFP by the 488 nm laser.
      ***Note:*** The acquired data were analyzed using FlowJo version 10 software.
  d. Aliquot the fibril preparation into sterile Protein LoBind tubes, snap-freeze in liquid nitrogen, and store at −80°C for further use.
    ***Note:*** The sample can remain stable at −80°C for up to 1 year.

## Expected outcomes

This protocol enables the robust production of endotoxin-free, full-length (2N4R) human recombinant WT tau protein suitable for use in immunocompetent cellular systems. When performed as described, the workflow yields milligram quantities of highly purified tau with negligible endotoxin contamination and preserved functional competence for downstream aggregation and seeding assays.

The WT 2N4R tau sequence is expressed as an N-terminal 6xHis-SUMO3 fusion from the pETM11-Sumo3-TauWT2N4R plasmid, enabling purification by IMAC with imidazole gradient elution followed by SENP2 protease-mediated tag removal. Starting from a 3 L bacterial culture, the IMAC step typically yields ∼35–40 mg of fusion protein (Figure 1). Cleavage of the 6×His-SUMO3 tag by SENP2 is efficient at enzyme-to-substrate ratios as low as 1:100 (w/w), generating full-length tau (∼60 kDa), which can be clearly resolved by SDS-PAGE (Figure 3).

During the IMAC rebind step, SENP2-cleaved tau does not consistently appear in the flow-through as expected for untagged proteins, but instead often remains associated with the column matrix. This behavior reflects the intrinsically disordered nature of tau, which promotes non-specific interactions with the resin under low-salt conditions.^28^^,^^34^^,^^35^ As a result, an imidazole gradient is typically required to recover cleaved tau from the column (Figure 4).

Subsequent cation exchange chromatography (CIEX) substantially improves purity by exploiting the net positive charge of tau at neutral pH. Full-length human 2N4R tau has a theoretical isoelectric point (pI) of approximately 8.2 and is therefore positively charged under the purification conditions used here, enabling efficient binding to the negatively charged cation exchange resin (Figure 6). Tau is subsequently eluted as a defined peak upon increasing ionic strength. Residual contaminants and truncated species may still be detectable, reflecting tau propensity to truncate^15^^,^^36^ but are significantly reduced at this stage. The final yield after CIEX and concentration is typically 3–4 mg of purified tau from a 3 L culture, with protein concentrations in the range of ∼1.5–1.6 mg/mL (Table 1).

A critical outcome of this protocol is the efficient removal of endotoxin. The combination of Triton X-114 on-column washing during IMAC and post-purification treatment with endotoxin removal resin consistently reduces endotoxin levels to ≤0.01 EU/mL at working concentrations (1 μg/mL tau) (Table 1). While total endotoxin levels in concentrated tau stocks may vary (typically ∼0.5–2.5 EU/mL depending on culture scale), dilution into experimental conditions ensures negligible activation of TLR4 signaling in microglia. This represents a substantial improvement over conventional purification approaches, where endotoxin contamination often confounds interpretation of immune responses.

The purified monomeric tau retains its ability to undergo heparin-induced fibrillization, forming β-sheet-rich fibrillar structures detectable by TEM and seeding bioassays (Figures 7C and 7D). However, fibril morphology (e.g., length, density, and degree of bundling) and seeding capacity in Tau RD P301S FRET biosensor assays may vary between preparations. Such variability is consistent with previous reports and reflects the intrinsic sensitivity of tau aggregation to subtle differences in protein quality and experimental conditions.^37^^,^^38^ For this reason, batch-specific quality control, including structural and functional validation, is recommended prior to downstream applications.

Overall, this protocol provides a reproducible method for generating endotoxin-free tau protein with defined biochemical and functional properties, enabling accurate investigation of tau-mediated effects in microglia and other immune-responsive systems without confounding inflammatory artifacts.

## Limitations

While this protocol enables reproducible, high-yield production of endotoxin-free recombinant WT 2N4R tau, several limitations should be considered when applying the method and interpreting downstream results.

As discussed above, batch-to-batch variability between preparations may occur, affecting overall yield, purity, and endotoxin levels. This may reflect differences in recombinant protein expression arising from factors such as bacterial starting material, together with protein recovery during downstream purification and the intrinsically disordered nature of tau, which can lead to non-specific interactions during chromatographic steps. As a result, rigorous quality control as outlined in the protocol is essential to ensure consistency across experiments.

Furthermore, binding of tau to the CIEX column is not always optimal, and partial loss of protein in the flow-through may be observed. This is influenced by residual imidazole carried over from IMAC, as well as buffer pH and ionic strength, which together determine tau binding efficiency. Although the conditions described here provide a reliable balance between yield and purity, further optimization of buffer composition or imidazole removal may improve recovery in specific settings.

Finally, while the heparin-induced tau aggregation outlines in this protocol provides a convenient, simple, scalable *in vitro* method for producing tau fibrils, it does not completely reproduce the structure and composition of tau aggregates *in vivo*. In particular, recombinant fibrils are structurally heterogeneous compared to brain-derived tau,^39^^,^^40^^,^^41^^,^^42^^,^^43^^,^^44^^,^^45^ differ in biochemical properties including resistance to chemical denaturation and proteolytic degradation,^44^ lack disease-associated post-translational modifications and cofactors which influence the protein’s biophysical properties, function, and aggregation^43^^,^^46^^,^^47^ and differ in seeding capacity and cellular effects.^41^ Where higher physiological relevance is required, seeding recombinant tau with disease-derived material may be considered.^48^^,^^49^^,^^50^

## Troubleshooting

### Problem 1

The French Press homogenizer becomes clogged, preventing uniform bacterial lysis (step 11).

### Potential solution

Large aggregates can sometimes be present in the bacterial suspension. Ensure the pellet is thoroughly resuspended in lysis buffer until no visible clumps remain. If necessary, pass the suspension through a cell strainer prior to loading onto the homogenizer.

### Problem 2

The bacterial lysate becomes highly viscous during homogenization, interfering with centrifugation and downstream purification (step 11).

### Potential solution

Viscosity is typically caused by the release of genomic DNA. Increase the concentration of DNase in the lysis buffer and ensure sufficient incubation time to promote DNA degradation.

### Problem 3

6xHis-SUMO3 tag cleavage is incomplete (step 22).

### Potential solution

SENP2 activity may be reduced by elevated salt or imidazole concentrations. Ensure that cleavage is performed in combination with dialysis to remove these components. Confirm that SENP2 has been properly stored and avoid repeated freeze-thaw cycles. If cleavage remains incomplete, optimize the enzyme-to-substrate ratio using a small-scale test. When using in-house purified SENP2, verify its activity prior to large-scale use.

### Problem 4

Sample loss occurs during dialysis (step 23).

### Potential solution

Sample loss is most commonly due to leakage from the dialysis tubing. Ensure the tubing is fully pre-wetted, free of defects, within its expiry date, and securely sealed at both ends. After loading the sample and applying the clips, hold the tubing upright for approximately 30–60 s and inspect for droplets forming at either clip or a noticeable decrease in the sample level before placing it into the dialysis buffer. Replace the tubing if leakage is observed.

### Problem 5

Low protein yield (step 37).

### Potential solution

Low yield may result from suboptimal expression or protein degradation. Use an appropriate expression strain such as BL21 (DE3), prepare fresh IPTG prior to induction, and include protease inhibitors in the lysis buffer. Perform all lysis steps at low temperature and ensure efficient cell disruption.

### Problem 6

High endotoxin levels in the final preparation (step 38).

### Potential solution

Insufficient endotoxin removal may arise from multiple steps, including inadequate Triton X-114 washing or reduced performance of the endotoxin removal resin. Increase the number of Triton X-114 washes during IMAC and ensure the buffer is maintained at 4°C, as temperature significantly influences endotoxin removal efficiency. When reusing HisTrap columns, perform thorough cleaning to prevent endotoxin accumulation. If necessary, extend the incubation time with the endotoxin removal resin or reapply the sample to the column. Ensure that the resin is properly regenerated if reused.

### Problem 7

Tau fibrils fail to form or show poor aggregation (steps 42–44).

### Potential solution

Fibrillization efficiency depends on heparin quality, molar ratio, and incubation conditions. Use freshly prepared, sterile-filtered heparin and maintain the specified 4:1 tau:heparin molar ratio. Use the appropriate shaking speed for the specific incubator, as differences in orbital diameter between instruments can affect aggregation kinetics. If fibril formation is incomplete, extend the incubation period and confirm fibrillization by TEM.

## Resource availability

### Lead contact

Further information and requests for resources and reagents should be directed to and will be fulfilled by the lead contact, Sally A. Cowley (sally.cowley@path.ox.ac.uk).

### Technical contact

Technical questions on executing this protocol should be directed to and will be answered by the technical contact, Maria Kreger Karabova (maria.karabova@gmail.com?).

### Materials availability

The plasmid pETM11SUMO3-TauWT2N4R used in this study has been deposited to Addgene (Teddington, UK; www.addgene.org, Cat#249347).

### Data and code availability

The published article contains all data generated or analyzed during this study.

## Acknowledgments

This work was supported by funding from The James Martin 21st Century Research Foundation (Partners in Tau). M.K.K. was supported by an Oxford E.P. Abraham Graduate Scholarship. The Sumo3-containing backbone of the plasmid used here was kindly provided by EMBL (pETM11-Sumo3-eGFP). We express our deep gratitude to Donna di Rienzo, Kourosh Ebrahimi, and Adam Harding for their valuable advice on protein purification; Abul Tarafder and Tanmay Bharat for access to Äkta Pure 25 and in-house purified SENP2 protein; Errin Johnson and Charlotte Melia for help in the Dunn School Electron Microscopy Facility; Robert Hedley and Vasiliki Tsioligka for help in the Don Mason Flow Cytometry Facility; and Rod Chalk from Centre for Medicines Discovery for protein mass verification. The graphical abstract was created with BioRender.com.

## Author contributions

Conceptualization, M.K.K., A.d.S.-B., W.S.J., and S.A.C.; methodology and writing, M.K.K. and A.d.S.-B.; supervision, W.S.J. and S.A.C.

## Declaration of interests

The authors declare no competing interests.
